# Trajectory Planner CDT-RRT* for Car-Like Mobile Robots toward Narrow and Cluttered Environments

**DOI:** 10.3390/s21144828

**Published:** 2021-07-15

**Authors:** Hyunki Kwon, Donggeun Cha, Jihoon Seong, Jinwon Lee, Woojin Chung

**Affiliations:** School of Mechanical Engineering, Korea University, Seoul 02841, Korea; fedaykin@korea.ac.kr (H.K.); donggeuncha@korea.ac.kr (D.C.); sjh0420@korea.ac.kr (J.S.); jwlee0623@korea.ac.kr (J.L.)

**Keywords:** mobile robot, motion control, trajectory generation, path planning, laser scanner, wheel odometry

## Abstract

In order to achieve the safe and efficient navigation of mobile robots, it is essential to consider both the environmental geometry and kinodynamic constraints of robots. We propose a trajectory planner for car-like robots on the basis of the Dual-Tree RRT (DT-RRT). DT-RRT utilizes two tree structures in order to generate fast-growing trajectories under the kinodynamic constraints of robots. A local trajectory generator has been newly designed for car-like robots. The proposed scheme of searching a parent node enables the efficient generation of safe trajectories in cluttered environments. The presented simulation results clearly show the usefulness and the advantage of the proposed trajectory planner in various environments.

## 1. Introduction

Recently, outdoor mobile robots have been receiving much attention because of the increased demand in the field of delivery and transportation. There are various wheel structures built according to target applications and environments. Two-wheel differential robots have been widely used owing to their simple structures. However, two-wheel robots have a lot of limitations in outdoor applications because it is difficult to overcome uneven ground conditions. Car-like robots are preferred in outdoor applications. The major drawback of car-like robots is that control problems become difficult due to nonholonomic constraints.

Path planning schemes are divided into two major groups. The first group includes search-based schemes and the second group includes sampling-based approaches. The search-based planner divides a space into small units such as grid-like graph structures or state lattice space as presented in [1]. Then, various search algorithms, including the Dijkstra’s algorithm, A* in [2] and D* in [3], can be applied to find solutions. Optimality can be obtained at the price of high computational cost. The search-based planner for various types of robots was proposed in [4,5]. The state lattice-based motion planning was applied not only to ground vehicles in [6], but also to aerial vehicles in [7].

The sampling-based planner extends trees or graph structures as shown in [8,9], respectively. Rapidly exploring Random Tree (RRT), shown in [10], is widely used because RRT may efficiently find feasible paths in high dimensional spaces with low computational cost as shown in [11,12,13]. Recently, RRT solutions in high dimensional spaces, such as temporal logic specifications, were proposed in [14].

Conventional RRTs showed many limitations mainly because the resultant RRT paths were generated without reflecting nonholonomic constraints. Recently, RRTs under the consideration of kinodynamic constraints have been proposed, as in [15,16,17,18,19].

There are two main issues in kinodynamic planning on the basis of RRTs. The first issue is to design proper sampling methods for the fast extension of random trees. In [20,21], sampling methods for RRT were proposed. The second issue is to define an appropriate distance metric for evaluating candidate paths. As Lavalle pointed out in [22], the distance metric considerably affects the performance of RRT planners. Euclidian distances in planes are commonly used when the fast expansion of the tree is significant. However, the consideration of kinodynamic constraints is essential in order to obtain efficient and feasible paths. Sampling in the control input space in [23] is one useful candidate. The planner in [23] requires an additional search algorithm in order to find the nearest neighbor nodes. The cost metric for informed RRT was proposed in [24].

As one of the outcomes of the Defense Advanced Research Projects Agency Urban Challenge, the closed loop Rapidly exploring Random Tree (CL-RRT) [25] was proposed. The CL-RRT generated reference guidelines through random sampling. CL-RRT generated feasible trajectories in the finite road region with the guidelines. CL-RRT mainly focused on autonomous vehicles in the road environment. A sampling technique was especially designed for roadways and parking lots.

The RRT* algorithm in [26] was proposed towards optimal path planning. A rewiring procedure of RRT* guaranteed asymptotic convergence to optimal solutions with a proper nearest range limit. Various planners based on RRT* were proposed in [27,28]. An RRT*-based planner was proposed for the high-speed maneuvering of autonomous vehicles in [23]. The scheme in [23] considered dynamic constraints including slip conditions. The distance metrics in [23] were designed for a relatively simple roadway without obstacles.

The Dual-Tree Rapidly exploring Random Tree (DT-RRT) was proposed for two-wheel differential mobile robots in [29]. DT-RRT utilized two separate trees with different distance metrics. DT-RRT efficiently generated feasible trajectories and local control inputs. A re-planning algorithm and tree updating process support real-time trajectory generation by reflecting the latest sensor information.

This paper aims to generate a feasible trajectory for car-like mobile robots in cluttered environments. Various planners were proposed for handling kinodynamic constraints or the fast extension of RRT. However, it is difficult to find the existing controller that is appropriate for a car-like robot in cluttered environments. Since the target application is the outdoor delivery robots, target environments are different from the environments of autonomous vehicles. Therefore, we propose the new path planning and motion control scheme CDT-RRT*, which is especially designed for car-like mobile robots. CDT-RRT* provides feasible trajectories and control inputs. CDT-RRT* can generate a kinodynamically feasible and goal-reachable trajectory with low computational cost. We propose a new parent searching algorithm of the dual-tree structure. The proposed replanning procedure includes re-propagation and tree-branching. Owing to the new searching algorithm, CDT-RRT* shows an outstanding performance in cluttered and narrow environments. The presented simulation results clearly demonstrate advantages over prior works.

The rest of this paper is organized as follows: Section 2 introduces the kinematic model and DT-RRT in [29]. The proposed CDT-RRT* scheme is presented in Section 3. The simulation results are presented in Section 4. Some concluding remarks are illustrated in Section 5.

## 2. The Kinematic Model and Dual-Tree RRT

### 2.1. Kinematic Model of a Car-Like Robot

**Figure 1:**
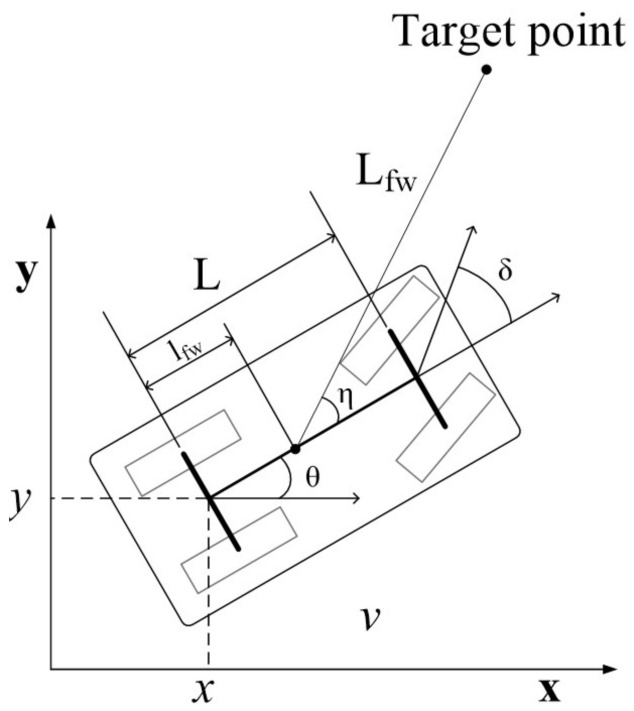
Geometry of the car-like mobile robot model.

The kinematic model of a car-like robot is shown in Figure 1. The reference point of the robot pose is on the center of the rear axle.

(1) $$\dot{x}=\left[\begin{array}{c} \dot{x}\\ \dot{y}\\ \dot{\theta }\\ \dot{\delta }\\ \dot{v} \end{array}\right]=\left[\begin{array}{c} vcos(\theta )\\ vsin(\theta )\\ \frac{v}{L}tan\left(\delta \right)\\ 0\\ 0 \end{array}\right]+\left[\begin{array}{c} 0\\ 0\\ 0\\ 0\\ 1 \end{array}\right]u_{1}+\left[\begin{array}{c} 0\\ 0\\ 0\\ 1\\ 0 \end{array}\right]u_{2}$$

Equation (1) is the kinematic model of car-like robots. The state vector $x={\left(x,y,\theta ,\delta ,v\right)}^{T}$ is a five-tuple that is composed of a robot pose, steering angle δ and translational velocity *v*. Control inputs are subject to the conditions $u_{1}\in \left[-a_{max},a_{max}\right]$ and $u_{2}\in \left[-\delta_{max},\delta_{max}\right]$.

The workspace is defined by $q\in R^{2}$ where q=(x,y). It is assumed that maps and obstacles are updated in real-time. The objective of the controller is to generate feasible trajectories $\tau :\left[0,T\right]\to C_{free}$ in finite time. The initial and goal conditions are given by τ(0)=x(0) and $\tau \left(T\right)\in B_{goal}$, respectively, whereas $B_{goal}=\left\{x|∥q_{goal}-x∥<\epsilon \right\}$. $L_{fw}$, $l_{\mathrm{fw}}$ and η are control parameters that will be explained in Section 3.1. The target point implies the desired target point for forward simulations.

### 2.2. Dual-Tree RRT

The Dual-Tree RRT (DT-RRT) was proposed in [29]. The dual-tree signifies the workspace tree and the state tree. The workspace tree is generated in 2D space q=(x,y). The distance metric is the Euclidean distance in a 2D plane. The workspace tree can be quickly extended by the node sampling technique that is similar to the conventional RRT. The state tree is generated in the state space $x={\left(x,y,\theta ,\delta ,v\right)}^{T}$. The cost is defined as the travel time of a candidate trajectory that is generated by reflecting the kinodynamic constraints of a robot. Therefore, smooth trajectories can be quickly generated by DT-RRT.

The workspace tree is extended by sampling new nodes. The state tree is extended by the utilization of newly created workspace tree nodes. During the extension, the lowest cost trajectory is selected out of many candidates. The quality of a trajectory is remarkably improved through the proposed parent node search and the reconnecting process.

The state node of DT-RRT has two node types including a stop state and a moving state. The stop state represents the state in which the robot has zero velocity, and a moving state represents the non-zero velocity. Four edge types of state trees generate a sequence of discrete motions, including turn-on-the-spot in cluttered environments. With the four edge types of DT-RRT, holonomic trajectories can be generated, and the probabilistic completeness of DT-RRT is guaranteed.

## 3. Car-like DT-RRT* (CDT-RRT*)

### 3.1. Motion Controller for Trajectory Generations

The major scope of this paper is to propose CDT-RRT* (Car-like DT-RRT*), which computes the control inputs of car-like mobile robots towards smooth and efficient motions. Two motion controllers in [30,31] were adopted in DT-RRT. Since the kinodynamic characteristics of car-like robots are completely different, motion controllers should be carefully redesigned.

The Pure-Pursuit Controller (PPC) in [32] was exploited as a local motion controller for CDT-RRT*. PPC has been widely used for the trajectory tracking of car-like robots and vehicles as shown in [25]. PPC does not guarantee that a robot can be steered to the desired target pose. Therefore, it is important to investigate whether PPC can be a useful local controller when target positions are given from the workspace tree. The control input of PPC in CDT-RRT* is redesigned as follows.

(2) $$\delta =-tan^{-1}\left(\frac{L\;sin\eta }{\frac{L_{fw}}{2}+l_{\mathrm{fw}}cos\eta }\right)$$

(3) $$v=min(v_{ref}-v_{c},a_{max}\cdot t_{cycle})$$

δ denotes a steering angle input. $L_{fw}$ is a look ahead distance. $l_{\mathrm{fw}}$ is the distance from the rear axle to the forward anchor point. η is the heading of the look-ahead point from the forward anchor point [25].

*v* denotes a velocity input of car-like robots. $v_{ref}$ is a reference linear velocity. Maximum speed trajectories will be generated when $v_{ref}=v_{max}$. $t_{cycle}$ is the cycle time of the controller.

The local controller computes control inputs through forward simulations. A local controller should satisfy three requirements. Firstly, the local controller should be able to cope with the changes in path length and velocity through appropriate parameter adjustment. The second requirement is that the final positioning error should be acceptably small. Finally, the positioning errors should converge through iterations.

**Figure 2:**
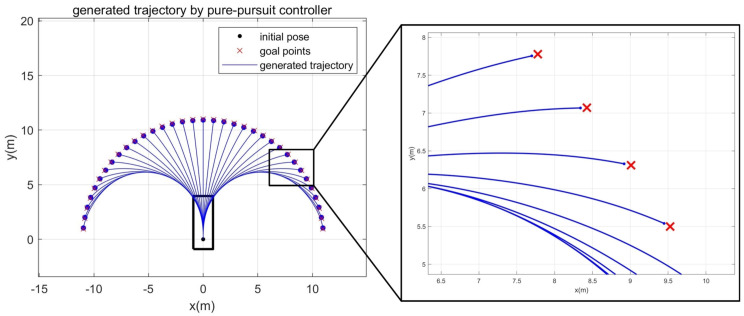
Trajectory generation results using Pure-pursuit controller.

In order to investigate whether PPC satisfies three requirements, simulations are carried out. Figure 2 shows the simulation results. The robot is driven to 35 target positions from the origin through the PPC. If the final positioning error is smaller than 10 cm, then the positioning is successful. We assume that the proposed method will be applied to outdoor delivery robots. The positioning accuracy of 10 cm is sufficient for an outdoor delivery robot because the human will handle the loading and unloading procedures. From Figure 2, it is clear that smooth trajectories are generated with small final positioning errors. Final positioning errors at all positions did not exceed 10 cm. The mean of the final positioning errors was 8.58 cm.

The state node of CDT-RRT* has two node types including a stop node and a moving node. The stop node is generated around the neighborhood of obstacles. The move node is generated in collision-free space. An edge type between two nodes is defined by the node type of a parent node and a child node.

There are four edge types in CDT-RRT*, including stop-move, stop-stop, move-move and move-stop. At each node, various different motion primitives can be selected according to the node types. By the selection of stop-move or stop-stop, CDT-RRT* generates a variety of trajectories that include stopping motions. This fact implies that rich behavior can be obtained by the sequential combination of piecewise smooth motions.

### 3.2. Dual-Tree Structure of CDT-RRT*

Tree extension puts an emphasis on fast-growing and tree restructuring plays a significant role in improving performance. Restructuring is composed of the parent node search and reconnect processes. The parent node search of the conventional DT-RRT started from the closest node to the newly created node $q_{new}$. Then the least cost trajectory was selected out of multiple candidate trajectories that were generated from parent nodes to $q_{new}$.

**Figure 3:**
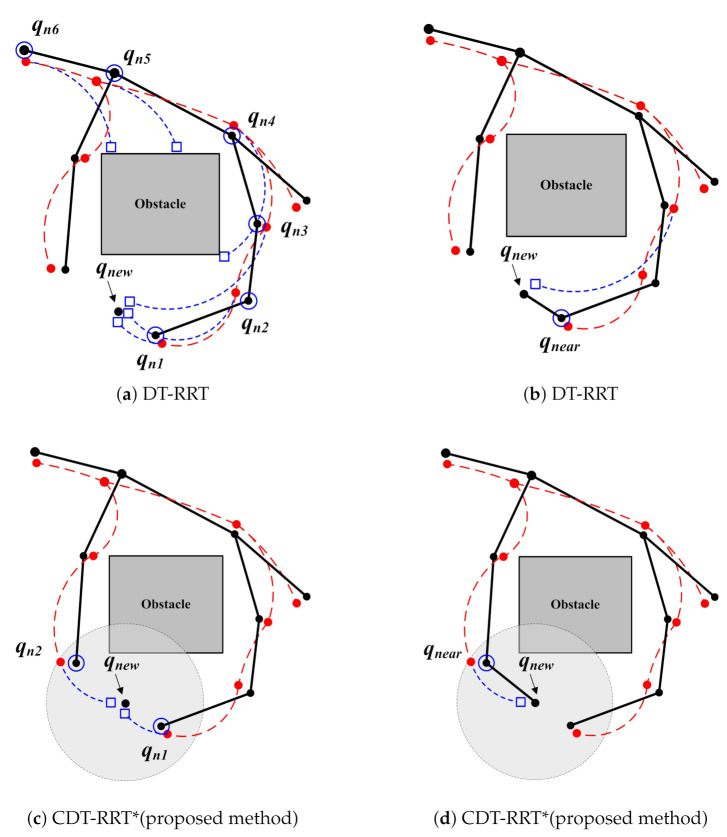
Finding parent node process in tree expansion.

Figure 3 illustrates the difference between the conventional DT-RRT and the proposed CDT-RRT*. Black trees are workspace trees. Dashed red lines show state trees. Once new node $q_{new}$ is created, the conventional DT-RRT connects between $q_{new}$ and parent nodes of closest node $q_{n1}$ as shown in Figure 3a. Nodes with blue circles in Figure 3a correspond to parent nodes. Figure 3b shows that the trajectory from $q_{n3}$ to $q_{new}$ was selected because of the lowest cost.

Although DT-RRT showed excellent performances in many practical applications, there are also limitations. One of the drawbacks is its limited restructuring capability. Since DT-RRT only selects the closest parent node, a better solution can be found according to the strategy of the parent node selection.

The proposed CDT-RRT* adopts multiple parent node candidates. As shown in Figure 3c, $Q_{nearlist}$ is defined as the close neighborhood of $q_{new}$ in the range of $B_{r}$. Then all nodes $Q_{nearlist}=\left\{q_{n1},q_{n2}\right\}$ in Figure 3c become parent node candidates. Finally, the minimum cost trajectory is selected out of all candidate trajectories from all parent nodes to $q_{new}$.

Algorithm 1 shows the proposed tree extension scheme. TW and TX are the workspace tree and the state tree. RandomSample() randomly picks a single point in 2D workspace (x,y). ExtWorkSpace$\left(\mathrm{TW},q_{rand}\right)$ finds $Q_{near}$, which is a nearest neighbor of $q_{rand}$. ExtWorkSpace$\left(\mathrm{TW},q_{rand}\right)$ connects $Q_{near}$ to $Q_{new}$. $Q_{new}$ is generated by $q_{rand}$ and has a position $q_{new}=\left(x_{new},y_{new}\right)$. The position of $q_{new}$ can be different from $q_{rand}$, because $q_{new}$ moved towards $Q_{near}$ by the maximum length of the tree extension. The subroutine NearNodeList$\left(Q_{new},B_{r}\right)$ finds the near nodes of $Q_{new}$ in the range of $B_{r}$. $\mathrm{TW}.\mathrm{Add}(Q_{near},Q_{new})$ implies that the workspace tree obtains a new child node $Q_{new}$. The parent node of $Q_{new}$ is $Q_{near}$. $\mathrm{TX}.\mathrm{Add}(X_{p},X_{min})$ implies that the state tree acquires new child node $X_{min}$. The parent node of $X_{min}$ is $X_{p}$. ReConnectTrees$\left(Q_{new},X_{min}\right)$ restructures the state tree after the addition of new nodes. Existing state nodes nearby $X_{min}$ are temporarily connected from $X_{min}$, while ReConnectTrees$\left(Q_{new},X_{min}\right)$ operates. If the cost of a temporarily connected path is lower than the original cost, the existing state node will be reconnected as a child of $X_{min}$.

Algorithm 2 describes the proposed strategy of selecting parent nodes for restructuring. Algorithm 2 returns minimum cost parent node $X_{p}$ after computing all trajectory costs from candidate nodes to $q_{new}$. GetStateNode$\left(Q_{near}\right)$ returns the state node that is derived from the workspace node $Q_{near}$. ExtendState$\left(q_{\mathrm{new}},X_{\mathrm{cur}},\mathrm{type}\right)$ generates a trajectory from $X_{cur}$ to $q_{new}$ in the range of $e_{r}$. The proposed motion controller in Section 3.1 is employed for trajectory generation. $q_{new}$ is the target point of the forward simulation in Figure 1.
**Algorithm 1** BuildTree*$\left(q_{0},x_{0},goal\right)$ $\mathrm{TW}.\mathrm{INIT}(q_{0},goal);$ $\mathrm{TX}.\mathrm{INIT}(x_{0},goal);$ **while** $t_{limit}$ **do**  $q_{rand}\leftarrow$ RandomSample();  $\left[Q_{new},Q_{near},type\right]\leftarrow$ ExtWorkSpace$\left(\mathrm{TW},q_{rand}\right);$  $Q_{nearlist}\leftarrow$ NearNodeList$\left(Q_{new},B_{r}\right)$  $\left[X_{p},X_{min}\right]\leftarrow$ FindParentState*$\left(Q_{nearlist},q_{new},type\right);$  **if** $X_{p}=nil$ **then**   continue;  **end if**  $\mathrm{TW}.\mathrm{Add}(Q_{near},Q_{new});$  $\mathrm{TX}.\mathrm{Add}(X_{p},X_{min});$  ReConnectTrees$(Q_{new},X_{min});$ **end while** return;

$B_{r}$ is a boundary of near nodes. It affects path quality and planning time. Large $B_{r}$ increases the number of candidate nodes. Therefore, the path quality can be improved and the computational cost of planning increases with the increase of $B_{r}$. Small $B_{r}$ may save planning time at the sacrifice of the path quality. In RRT* [26], a scheme that dynamically decreased $B_{r}$ was proposed. The asymptotic optimality of RRT-based planning with decreasing $B_{r}$ was shown in [26]. The determination of $B_{r}$ in CDT-RRT* exploited a similar approach of the conventional RRT*.

(4) $$\begin{array}{c} B_{r}=min\left\{\gamma_{RRT^{*}}{\left(log\left(card\left(V\right)\right)/card\left(V\right)\right)}^{1/d},\eta \right\}\\ \gamma_{RRT^{*}}=2{\left(1+1/d\right)}^{1/d}{\left(\mu \left(X_{free}\right)/\zeta_{d}\right)}^{1/d} \end{array}$$

**Algorithm 2** FindingParentState*$\left\{Q_{nearlist},q_{new},type\right\}$ $X_{p},X_{min}\leftarrow nil;$ $cost_{min}\leftarrow inf;$ **for all** $Q_{near}$ ∈ $Q_{nearlist}$ **do**  $X_{cur}\leftarrow$ GetStateNode$\left(Q_{near}\right);$  $\left[X_{new},e_{r},cost\right]\leftarrow$ ExtendState$\left(q_{\mathrm{new}},X_{\mathrm{cur}},\mathrm{type}\right)$  **if** $e_{r}\leq B_{r}\;\mathrm{and}\;cost\leq cost_{min}$ **then**   $\left[X_{p},X_{min},cost_{min}\right]\leftarrow \left[X_{cur},X_{new},cost\right];$  **end if** **end for** $\mathrm{return}\;X_{p},X_{min};$

As described in [26], card(V) is a cardinal number of workspace tree size. *d* is a dimension of the workspace, which is two in CDT-RRT*. $\mu (X_{free})$ denotes the Lebesgue measure of the obstacle-free space, and $\zeta_{d}$ is the volume of the unit ball in the d-dimensional Euclidean space. η is provided by the local steering function, which is ExtendState in CDT-RRT*. η of CDT-RRT* is 3 × the maximum length of the tree extension (which is 3 m).

In Figure 3c, the proposed CDT-RRT* selects $q_{n2}$ in addition to $q_{n1}$ as the parent node candidates. DT-RRT selected only $q_{n1}$ and parent nodes of $q_{n1}$ under the same condition as shown in Figure 3a. Figure 3d shows the completely different trajectory of the proposed CDT-RRT*. The resultant trajectory cost of CDT-RRT* is significantly lower than the cost of the DT-RRT trajectory in Figure 3b. The major advantage of CDT-RRT* is that restructuring is carried out with a wide variety of diverse candidate trajectories. Therefore, CDT-RRT* shows superior performances over prior works, especially in narrow and cluttered environments, because the minimum cost trajectory can be efficiently found from many trajectories with different homotopy.

CDT-RRT* includes replanning procedures for practical trajectory following. The replanning algorithm carries out translation of the existing tree to deal with localization error and disturbance of the controller. After translation of the whole tree, the lowest cost trajectory to the goal is selected. Then, collision checking for the lowest cost trajectory is carried out. If any collision occurs due to a map update or translation of the tree, unreachable nodes due to the collision are deleted. Then, the remaining orphan nodes are reconstructed based on the workspace tree. This reconstruction possibly saves planning time owing to the conservation of orphan nodes. The branching tree sequence is carried out after reconstruction. While branching the tree, the root node of the tree is generated as the robot moves forward.

Table 1 shows the qualitative comparison among kinodynamic planners. Six significant considerations are summarized towards kinodynamic planning in cluttered environments. Table 1 clearly visualizes unsolved problems and the differences among controllers. It is clear that the proposed CDT-RRT* is advantageous from a variety of aspects. CDT-RRT* can handle the kinodynamic constraints of car-like robots and generate piecewise smooth motions. By sampling in the $R^{2}$ configuration space, CDT-RRT* could extend the tree rapidly with goal bias. The proposed parent node selection and re-connect scheme greatly contribute to finding asymptotic optimal trajectories in cluttered environments. The proposed replanning scheme can maintain the dual-tree structure by conserving orphan nodes while robots keep moving and updating sensor information.

## 4. Simulation Results

Seven simulations are carried out for verification. Simulation conditions are listed in Table 2.

**Figure 4:**
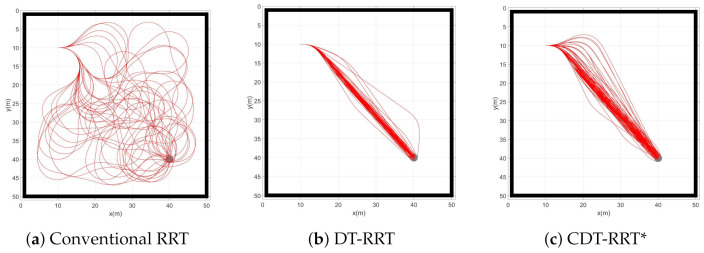
Resultant trajectories from simulation 1.

Figure 4 shows the result of simulation 1. Boundary conditions of simulation 1 were initial condition $x_{s}=\left(10,10,0,0,0\right)$ and goal $q_{g}=\left(40,40\right)$. The environment was free of obstacles. Three schemes were tested for comparison. One was the conventional RRT, the second was the conventional DT-RRT and the third was the proposed CDT-RRT*. Each scheme tried to generate 100 trajectories. If a trajectory could not be generated in 30 s, the trial was counted as a failure. In Table 3, the computing time indicates the required time to compute a single trajectory. The travel time indicates the moving time along the trajectory from the start to the goal. The failure count signifies the number of the failed trials out of 100 trajectory generations.

The conventional RRT was implemented for comparison. The conventional RRT sampled a random point in the 2-dimensional configuration space. The sampled point and the nearest node of the sampled point were connected by *ExtendState* of CDT-RRT*. If the connection fails, the conventional RRT samples another point until finding a feasible path. The conventional RRT showed a significantly low success rate of node extension.

From Table 3, it can be seen that the success rate of RRT trajectory generation was only 34%. The travel time and computing time were much longer than those of DT-RRT or CDT-RRT*. In the case of a car-like robot, the limitation of steering angles limits the motion of the robot. Therefore, most of the newly created RRT nodes become infeasible because new nodes were generated without consideration of kinematic constraints. It is desirable to obtain smooth and efficient trajectories from the viewpoint of quality. From Figure 4a, it is clear that the quality of RRT trajectories is poor. The poor performance of RRT was caused by the lack of restructuring processes.

Table 3 shows that DT-RRT and CDT-RRT* successfully generated 100 trajectories without any failure. This result was made possible owing to the application of local controllers where kinodynamic constraints were considered. From the viewpoint of trajectory quality, there were no significant differences between DT-RRT and CDT-RRT* in the obstacle-free environment. Figure 4b,c show that the two schemes have similar performances and provide satisfactory results.

**Figure 5:**
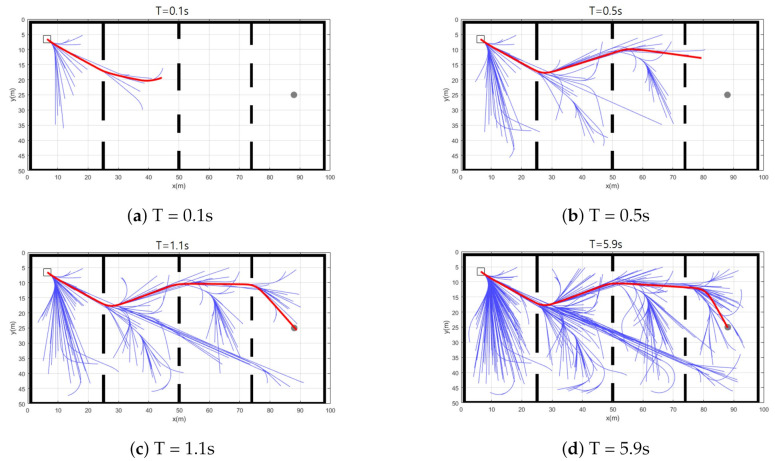
Resultant trajectories of CDT-RRT* from simulation 2.

**Figure 6:**
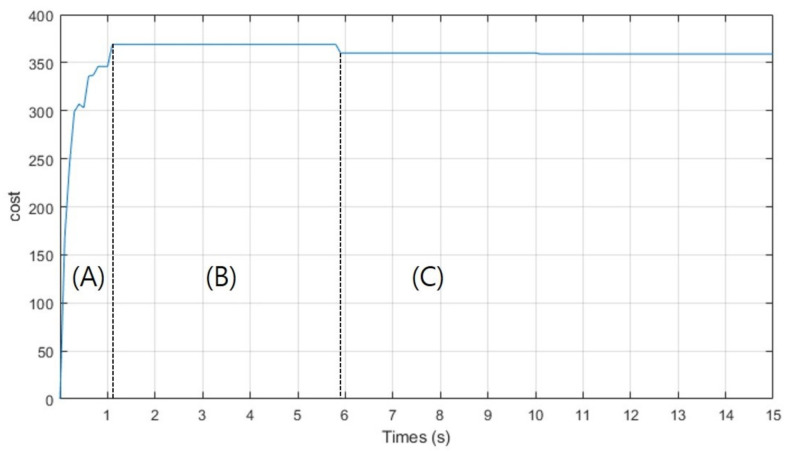
The cost of the solution path by CDT-RRT* in simulation 2.

Simulation 2 was carried out to investigate the trajectory expansion of CDT-RRT*. Figure 5 shows the planning result of CDT-RRT* in simulation 2. The blue lines show the state tree of CDT-RRT*. The thick red line indicates the solution path. The grey circle describes the goal region. CDT-RRT* returns the path nearest to the goal until securing a goal-reachable path as shown in Figure 5a,b. The cost of the solution path increased during that period as shown in zone (A) of Figure 6. After securing a goal-reachable path as in Figure 5c, the tree of CDT-RRT* kept extending and searching for a better solution, as shown in zone (B). If CDT-RRT* finds a lower-cost solution, the cost decreases as shown in zone (C).

**Figure 7:**
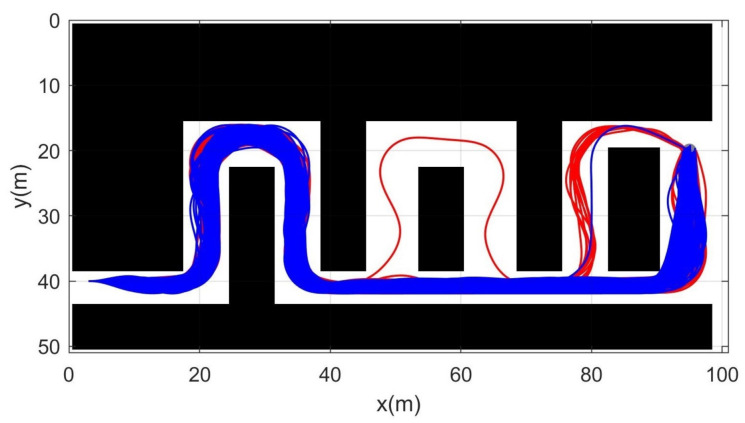
Resultant trajectories from simulation 3. Red lines: DT-RRT, Blue lines: CDT-RRT*.

**Figure 8:**
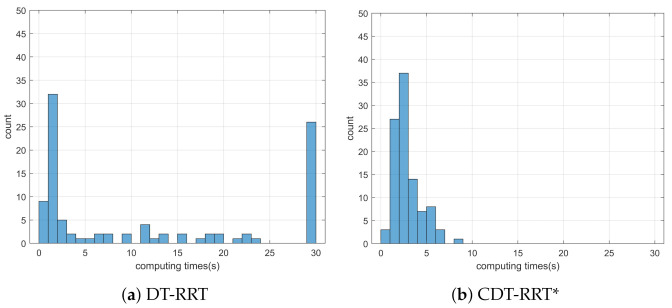
Histogram of the computing time for the results from simulation 3.

Simulation 3 was carried out in the consecutive U-shaped corridor environment as shown in Figure 7. In simulation 2, the robot was driven from $x_{s}=\left(3,40,0,0,0\right)$ to $q_{g}=\left(95,20\right)$. Table 4 shows the quantitative results of the comparison. It can be seen that RRT could not generate trajectories in most cases. Therefore, RRT is inapplicable in the cluttered environment, as in Figure 7. It is noteworthy that the success rate of CDT-RRT* trajectory generation was 100%, while the success rate of DT-RRT was only 74%. In addition, the computing time of the trajectories can be remarkably saved by the use of CDT-RRT*. The mean computing time of CDT-RRT* is only 23% of DT-RRT’s computing time. Figure 8 shows the histograms of the computing times for DT-RRT and CDT-RRT*. It can be seen that solutions can be obtained in a short time for the case of CDT-RRT*. From the result, it is evident that the proposed CDT-RRT* shows superior performances in a cluttered environment owing to its excellent restructuring capability. The high success rate of trajectory generation and reduced computing cost enable real-time computation and high-speed obstacle avoidance motions.

**Figure 9:**
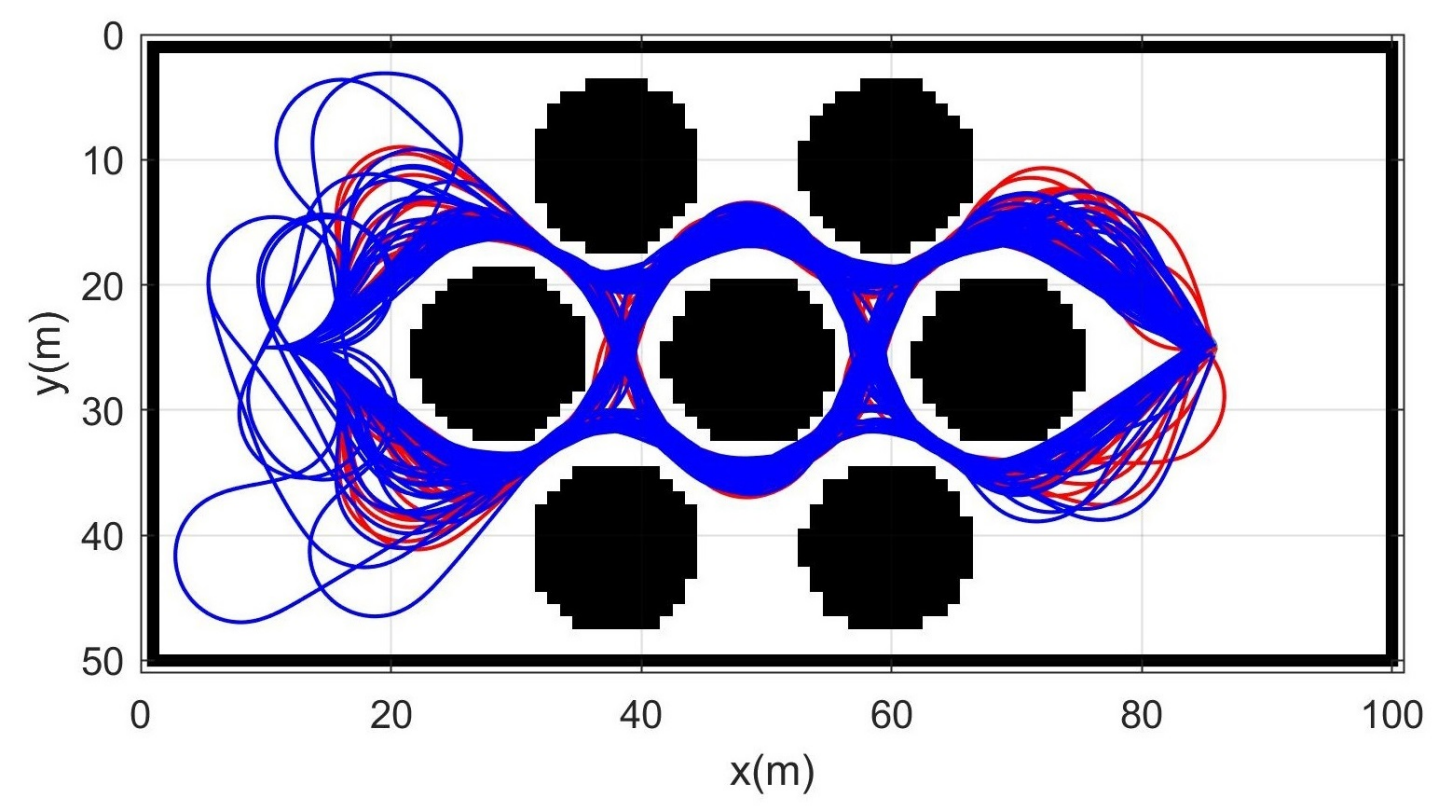
Resultant trajectories from simulation 4. Red lines: DT-RRT, Blue lines: CDT-RRT*.

**Figure 10:**
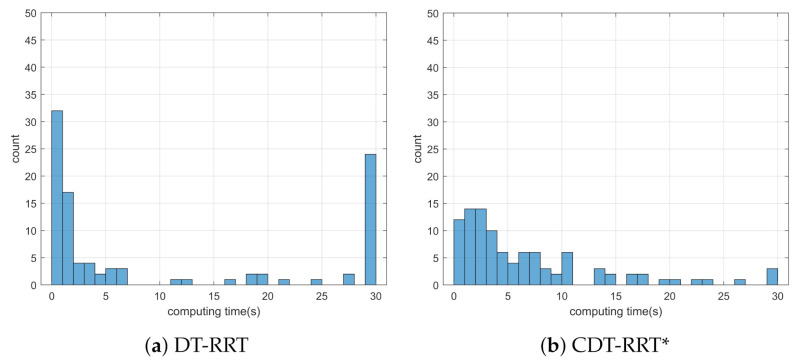
Histogram of the computing time for the results from simulation 4.

Simulation 4 was carried out in the cluttered narrow environment as shown in Figure 9. The robot was driven from $x_{s}=\left(10,25,0,0,0\right)$ to $q_{g}=\left(85,25\right)$. Table 5 shows the result of a quantitative comparison among the three schemes. It is obvious that RRT fails to generate feasible trajectories because the success rate of trajectory generation is only 1%. The failure rate of DT-RRT is 23% and it is much higher than the 3% failure rate of CDT-RRT*. The computing time of DT-RRT was 50% longer than that of CDT-RRT*. Figure 10 shows the histograms of the computing times for DT-RRT and CDT-RRT*. It can be seen that we can expect quick and stable performances for CDT-RRT* in most trials. Although the travel time of the two schemes did not show a significant difference, the difference in terms of the computing time and the success rate greatly affects the real-time performance of robot navigation.

**Figure 11:**
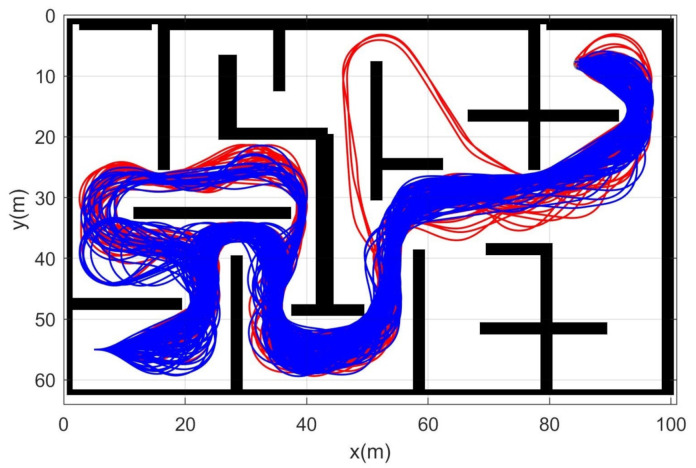
Resultant trajectories from simulation 5. Red lines: DT-RRT, Blue lines: CDT-RRT*.

**Figure 12:**
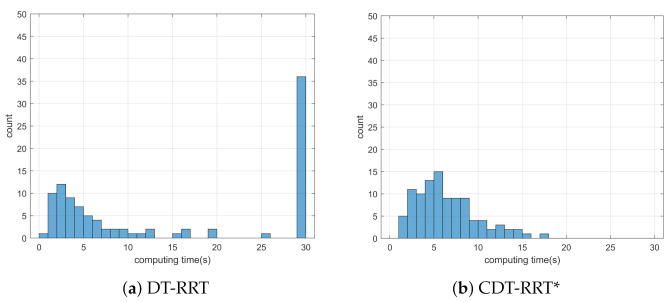
Histogram of the computing time for the results from simulation 5.

Simulation 5 was carried out in the maze environment as shown in Figure 11. The robot was driven from $x_{s}=\left(5,55,0,0,0\right)$ to $q_{g}=\left(85,5\right)$. Table 6 shows the comparative results among the three schemes. RRT showed complete failure in the long cluttered environment. The proposed CDT-RRT* did not fail to find the trajectory in any trial, while the failure rate of DT-RRT was 36%. The mean computing time of DT-RRT was 2.2 times longer than that of CDT-RRT*. Figure 12 shows the histograms of the computing times for DT-RRT and CDT-RRT*. It is evident that all CDT-RRT* trajectories were obtained in a short time, while DT-RRT showed excessive computing time and unstable performances in many trials. From the viewpoint of the mean travel time, CDT-RRT* took only 66 s, while DT-RRT took 72 s—10% longer than that of the proposed method.

**Figure 13:**
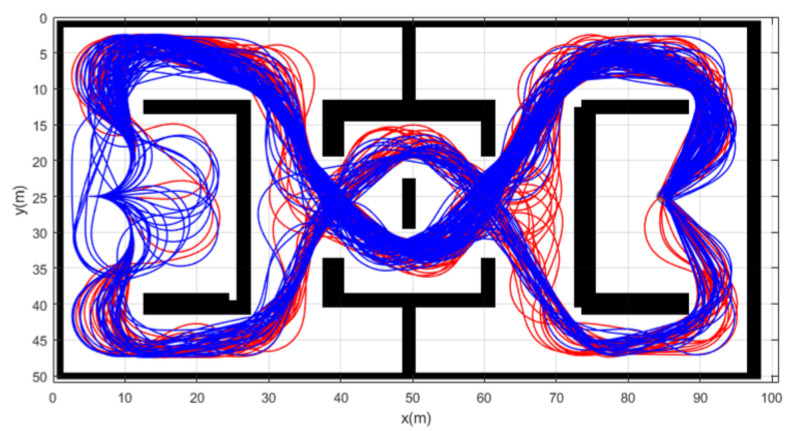
Resultant trajectories from simulation 6. Red lines: DT-RRT, Blue lines: CDT-RRT*.

**Figure 14:**
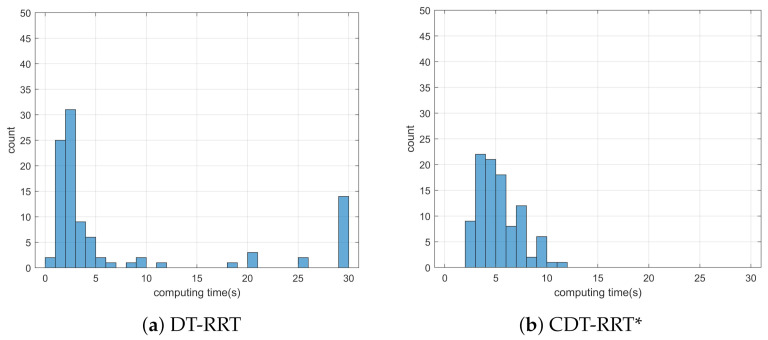
Histogram of the computing time for the results from simulation 6.

Simulation 6 was carried out in a cluttered environment as shown in Figure 13. The robot was driven from $x_{s}=\left(5,25,0,0,0\right)$ to $q_{g}=\left(85,25\right)$. Table 7 shows the comparative results among the three schemes. RRT showed complete failure in the long cluttered environment. The proposed CDT-RRT* did not fail to find the trajectory at any trial, while the failure rate of DT-RRT was 14%. The mean computing time of DT-RRT was 1.4 times longer than that of CDT-RRT*. Figure 14 shows the histograms of the computing times for DT-RRT and CDT-RRT*. It is evident that all CDT-RRT* trajectories were obtained in a short time, while DT-RRT showed excessive computing time and unstable performances in many trials. From the viewpoint of the mean travel time, CDT-RRT* took only 61 s, while DT-RRT took 62 s. Although the travel time of the two schemes did not show a significant difference, the difference in terms of the computing time and the success rate greatly affects the real-time performance of robot navigation.

**Figure 15:**
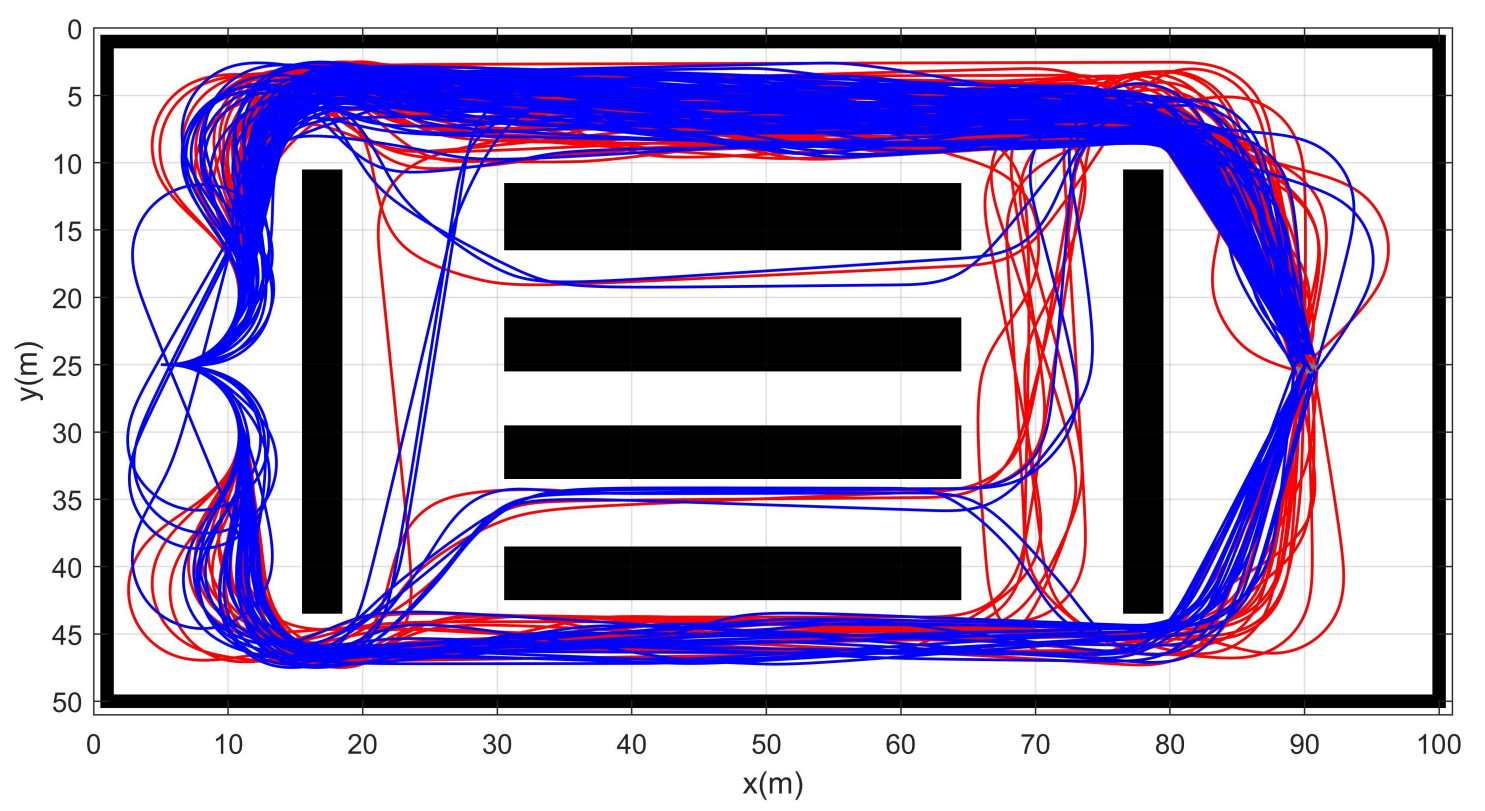
Resultant trajectories from simulation 7. Red lines: DT-RRT, Blue lines: CDT-RRT*.

**Figure 16:**
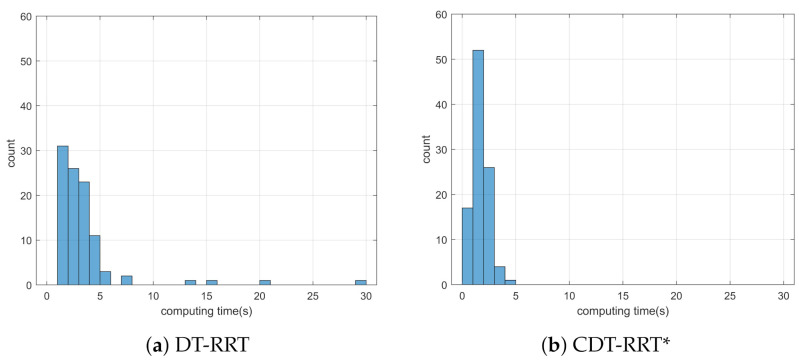
Histogram of the computing time for the results from simulation 7.

Simulation 7 was carried out in a narrow environment as shown in Figure 15. The robot was driven from $x_{s}=\left(5,25,0,0,0\right)$ to $q_{g}=\left(90,25\right)$. Table 8 shows the comparative results among the three schemes. RRT showed complete failure in the long cluttered environment. The proposed CDT-RRT* did not fail to find the trajectory in any trial, while the failure rate of DT-RRT was 1%. The mean computing time of DT-RRT was 2.0 times longer than that of CDT-RRT*. Figure 16 shows the histograms of the computing times for DT-RRT and CDT-RRT*. It is evident that all CDT-RRT* trajectories were obtained in a short time, while DT-RRT showed excessive computing time and unstable performances in many trials. From the viewpoint of the mean travel time, CDT-RRT* took 46 s, while DT-RRT took 47 s. Although the travel time of the two schemes did not show a significant difference, the difference in terms of the computing time and the success rate greatly affects the real-time performance of robot navigation.

Therefore, it can be concluded that the proposed CDT-RRT* generates feasible trajectories with short travel times. In addition, the proposed parent search algorithm shows outstanding performances especially in narrow cluttered environments where conventional schemes show limited performances.

## 5. Conclusions

This paper proposed a new trajectory planner, CDT-RRT*, for car-like mobile robots. CDT-RRT* utilizes the workspace tree and the state tree for the fast-growing of trees as well as for kinodynamically feasible trajectory generation. The proposed restructuring scheme provides outstanding performances over prior works owing to its superior search capability out of diverse candidate trajectories. CDT-RRT* is especially useful when the robot moves in narrow cluttered environments. Owing to the appropriate design of the tree search algorithm, a high success rate of trajectory generation and a reduced computing time have been achieved. Those two advantages greatly contribute to real-time applications. The presented simulation results clearly show the advantage of the proposed CDT-RRT* in various environments.

Although it is possible to generate the maneuvering, including both forward and backward motions, CDT-RRT* may fail to generate feasible trajectories in extremely narrow and cluttered environments. Future works will include the development of different local controllers or improved node sampling strategies in order to overcome extreme situations.

## Figures and Tables

**Table 1:** The comparison of kinodyamic trajectory planners.

	RRT [10]	CL-RRT [25]	High Speed RRT* [23]	DT-RRT [29]	CDT-RRT*
Car-like model	None	Good	Good	None	Good
Piecewise motion	None	None	None	Good	Good
Goal-biased sampling	Good	Fair	Fair	Good	Good
Cluttered Environments	Good	Fair	Fair	Fair	Good
Replanning	None	Fair	Fair	Good	Good
Saving orphan nodes	None	None	None	Good	Good

**Table 2:** The simulation conditions.

Parameters	Values		Specifications
$v_{max}$	2.7 m/s	CPU	Intel i7-2620M 2.7 Ghz
$a_{max}$	1.8 m/s${}^{2}$	RAM	4 GB (1333 MHz)
L	2.8 m	OS	Ubuntu 11.04
$\left[\delta_{min},\delta_{max}\right]$	$\left[-30^{\circ },30^{\circ }\right]$	Middleware	Robot Operating System
${\dot{\delta }}_{max}$	$20^{\circ }/$s${}^{2}$	Language	C++

**Table 3:** Summary of Planning Results from simulation 1.

		Mean (s)	σ	Min (s)	Max (s)	Failure Count
RRT	Computing time	8.62	7.93	0.02	27.59	66
	Travel time	35.01	7.15	19.20	49.80	
DT-RRT	Computing time	0.36	0.20	0.06	1.04	0
	Travel time	18.67	0.62	17.30	19.30	
CDT-RRT*	Computing time	0.36	0.52	0.07	5.00	0
	Travel time	18.87	0.82	17.30	21.70	

**Table 4:** Summary of Planning Results from simulation 3.

		Mean (s)	σ	Min (s)	Max (s)	Failure Count
RRT	Computing time	29.89	1.11	18.90	30.00	99
	Travel time	85.50	0.00	85.50	85.50	
T-RRT	Computing time	12.08	12.19	0.60	30.00	26
	Travel time	58.79	2.22	55.70	73.30	
CDT-RRT*	Computing time	2.79	1.47	0.70	8.60	0
	Travel time	57.91	1.07	55.70	61.30	

**Table 5:** Summary of Planning Results from simulation 4.

		Mean (s)	σ	Min (s)	Max (s)	Failure Count
RRT	Computing time	29.81	1.93	10.71	30.00	99
	Travel time	62.70	0.00	62.70	62.70	
DT-RRT	Computing time	10.20	12.30	0.20	30.00	23
	Travel time	40.56	1.91	35.70	45.20	
CDT-RRT*	Computing time	6.78	7.03	0.32	30.00	3
	Travel time	40.71	4.71	35.20	61.30	

**Table 6:** Summary of Planning Results from simulation 5.

		Mean (s)	σ	Min (s)	Max (s)	Failure Count
RRT	Computing time	30.00	-	30.00	30.00	100
	Travel time	-	-	-	-	
DT-RRT	Computing time	14.50	12.39	0.98	30.00	36
	Travel time	72.83	11.60	58.30	102.70	
CDT-RRT*	Computing time	6.46	3.44	1.60	17.34	0
	Travel time	66.54	8.52	57.60	86.00	

**Table 7:** Summary of Planning Results from simulation 6.

		Mean (s)	σ	Min (s)	Max (s)	Failure Count
RRT	Computing time	30.00	-	30.00	30.00	100
	Travel time	-	-	-	-	
DT-RRT	Computing time	7.61	10.03	0.90	30.00	14
	Travel time	62.40	3.37	57.50	74.60	
CDT-RRT*	Computing time	5.25	2.06	2.20	11.10	0
	Travel time	61.33	4.22	56.00	75.70	

**Table 8:** Summary of Planning Results from simulation 7.

		Mean (s)	σ	Min (s)	Max (s)	Failure Count
RRT	Computing time	30.00	-	30.00	30.00	100
	Travel time	-	-	-	-	
DT-RRT	Computing time	3.53	3.78	1.23	30.00	1
	Travel time	47.10	5.19	42.60	66.90	
CDT-RRT*	Computing time	1.74	0.69	0.60	4.45	0
	Travel time	46.43	4.29	42.60	61.80	
